# Continuous Glucose Monitoring of Glycemic Variability During Fasting Post-Sleeve Gastrectomy

**DOI:** 10.1007/s11695-020-04505-4

**Published:** 2020-07-17

**Authors:** Ebaa Al-Ozairi, Abeer El Samad, Jumana Al Kandari, Etab Taghadom, Safwaan Adam, Carel le Roux, Akheel A. Syed

**Affiliations:** 1 Clinical Research Unit, Medical Division, Dasman Diabetes Institute, P.O. Box 1180, 15462 Dasman, Kuwait; 2 Faculty of Medicine, Kuwait University, Kuwait City, Kuwait; 3 Department of Diabetes, Endocrinology and Obesity Medicine, Salford Royal NHS Foundation Trust and University Teaching Hospital, Salford, Manchester, UK; 4 Faculty of Biology, Medicine and Health, University of Manchester, Manchester, UK; 5 Diabetes Complications Research Centre, University College Dublin, Dublin, Ireland

**Keywords:** Fasting, Continuous glucose monitoring system, Bariatric surgery, Sleeve gastrectomy, Type 2 diabetes

## Abstract

**Introduction:**

Day-long fasting creates considerable metabolic stress that poses challenges in people with diabetes and those who have undergone bariatric surgery. Clinical knowledge of glucose fluctuations and the risks for such patients during fasting is limited.

**Objectives:**

This study examined the effect of intermittent fasting on glucose excursions, hypoglycemia, and hyperglycemia in people with or without diabetes who had sleeve gastrectomy compared with healthy individuals.

**Methods:**

This open-label, prospective study compared interstitial glucose profiles measured with continuous glucose monitoring system for 72 h during fasting and non-fasting periods between four groups comprising 15 participants each: people with obesity and medicine-treated type 2 diabetes (T2D) only, obesity and T2D treated with sleeve gastrectomy, obesity without T2D treated with sleeve gastrectomy, and healthy, normal-weight non-diabetic controls.

**Results:**

The mean 72-h glucose concentration was significantly lower during the fasting period for all groups (*p* ≤ 0.041), with the highest glucose concentrations in the medicine-treated T2D-only group and the lowest concentrations in the sleeve gastrectomy in non-T2D group. The mean glucose profiles of all the groups showed a marked increase in interstitial glucose on breaking the fast, which was exaggerated in the two diabetes groups. The mean amplitude of glycemic excursions did not differ significantly within each group between fasting and non-fasting. No significant difference was noted in the fraction of time in the hypoglycemic range between the fasting and non-fasting periods in any group.

**Conclusion:**

Intermittent fasting had no adverse effect on glycemic control in people with or without diabetes who had undergone sleeve gastrectomy.

**Electronic supplementary material:**

The online version of this article (10.1007/s11695-020-04505-4) contains supplementary material, which is available to authorized users.

## Introduction

Many people undertake fasting for lifestyle reasons (e.g., intermittent fasting diets) or religious rituals (e.g., the Christian season of Lent, the Islamic month of Ramadan, Jewish days of fasting such as Yom Kippur, and the Hindu month of Shravan). In recent years, intermittent fasting has gained popularity because of its beneficial effects on metabolic and glycemic control, and insulin sensitivity [1, 2]. A high visceral fat content reduces insulin sensitivity and glucose uptake in people with obesity and type 2 diabetes (T2D) [3]. The weight loss and reduction in visceral fat during intermittent fasting can reverse these effects, thus increasing insulin sensitivity and reducing glycemia [4]. Adiponectin levels increase during intermittent fasting [5], thereby modulating insulin activity and improving beta-cell function [6]. Significant weight loss can help to place T2D into remission [7]. Evidence also suggests that intermittent fasting has other health benefits, including reducing the risk of developing diabetes, hypertension, hyperlipidemia, hepatic steatosis, and coronary artery disease [8]. Thus, professional guidelines include intermittent fasting in the management and prevention of diabetes-related complications [9].

Although lifestyle, dietary, and behavioral changes remain first-line strategies for managing obesity, bariatric surgery is an effective and popular treatment globally [10]. Bariatric surgery can achieve sustained weight loss leading to improvements in several obesity-related conditions, including T2D, hyperlipidemia, hypertension, and obstructive sleep apnea [11], and is reported to be a cost-effective treatment for obesity [12]. However, little is known of the effects of fasting in people who have undergone bariatric surgery.

Among the few clinical studies investigating intermittent fasting in people with T2D, the reported health benefits are inconsistent [13]. The fasting time (hours), duration (days), and energy intake vary across these studies. The Islamic ritual fasting during the month of Ramadan follows an intermittent fasting procedure, lasting from predawn to sunset, with complete abstention from eating or drinking. A multicenter study reported that diurnal fasting with no drink or food has an increased incidence of hypoglycemia in patients with T2D [14]. In a similar study, the rate of hypoglycemia during fasting periods was higher compared with non-fasting periods [15]. However, some studies have found that fasting improves glycemic control [16, 17]. Therefore, the safety of fasting in individuals with diabetes is of concern to both patients and healthcare professionals [18].

The aim of our study was to investigate the safety of intermittent fasting by examining fluctuations in glucose levels in people with diabetes, with or without a history of sleeve gastrectomy, during fasting and non-fasting periods compared with healthy controls using a continuous glucose monitoring system (CGMS) [19].

## Methods

We carried out an open-label, prospective, non-interventional observational study of the effects of fasting on glucose profiles in diabetic and non-diabetic patients treated with or without sleeve gastrectomy compared with healthy controls.

### Participants and Study Design

The study included 4 groups: (1) people with obesity and medicine-treated T2D (diabetes-only, DO), (2) T2D and obesity treated with sleeve gastrectomy (sleeve gastrectomy and diabetes, SGD), (3) obesity without diabetes treated with sleeve gastrectomy (sleeve gastrectomy only, SGO), and (4) healthy controls (HC). Diagnosis of T2D was based on the American Diabetes Association criteria [20], and remission of diabetes after surgery as per criteria recommended by Buse et al. [21]. The surgical participants had undergone sleeve gastrectomy at least 12 months prior to recruitment. SGD participants who did not go into remission of diabetes after the surgery were taking conventional medication for T2D. CGMS was conducted for 72 h (3 consecutive days) during Ramadan (fasting period) and for 72 h post-Ramadan (non-fasting period). The fasting period included daylight hours, from predawn to sunset (approximately 16 h), during which participants completely abstained from eating, drinking, or oral medications. The primary outcome measure was mean 72 h interstitial glucose concentration in fasting period vs. non-fasting period. We also compared the fraction of time the blood glucose concentrations were in the ranges of hypoglycemia and hyperglycemia, the mean amplitude of glycemic excursion (MAGE), and glycated hemoglobin (HbA1c) concentrations with and without fasting between the groups.

### Sample Size

As this was a novel study with no previous similar data to advise power/sample size calculations, we used empirical sample sizes aimed at the primary endpoint. Thus, we recruited 15 participants into each of the study and control groups.

### Inclusion Criteria

Patients were recruited from our bariatric and diabetes registries. Those who were residents of Kuwait aged ≥ 21 years were eligible to participate in this study. These potential participants were contacted to seek their willingness to undertake CGMS during fasting and non-fasting periods. All participation was voluntary, and written informed consent was obtained. Participants had to be willing to use CGMS for a minimum duration of 3 consecutive days during fasting and non-fasting periods. They were also required to check their finger-prick capillary blood glucose 4 to 6 times per day and record their meals, medications, and exercise on a standardized log sheet with full instructions.

### Exclusion Criteria

The study excluded patients with type 1 diabetes mellitus, poor glycemic control (HbA1c > 10%), severe active diabetes complications, elevated liver enzyme concentrations, pregnancy or breast feeding, a history of hospitalization within the preceding 3 months, and hypoglycemia unawareness (documented blood glucose < 3 mmol/L without hypoglycemic symptoms).

### Measures

All participants underwent baseline assessments: a review of health issues and medications and measurement of weight, height, and body mass index (BMI), blood pressure, and blood tests, including concentrations of glucose, insulin, HbA1c, and cholesterol at baseline (prefasting) and postfasting (nonfasting) periods. The extent of glucose variability during hypoglycemia and hyperglycemia was compared.

### Continuous Glucose Monitoring System

Participants received training in the use of a Medtronic 4th generation iPro 2 CGMS with an Enlite sensor (Medtronic MiniMed, Inc., Northridge, CA). The sensor, inserted subcutaneously into the anterior abdominal wall, measured interstitial glucose every 10 s, with storage of the average every 5 min. Each participant was asked to return after 72 h for sensor disconnection and downloading of the data using Medtronic Carelink software for diabetes. The monitoring process was repeated during the non-fasting phase. We defined a hypoglycemic episode on the CGMS as a single interstitial glucose concentration < 3.9 mmol/L with or without symptoms and hyperglycemia as a single interstitial glucose concentration > 10.0 mmol/L. Each participant was asked to measure capillary glucose at least 4 times per day using a Contour Glucometer provided by the study team.

### Statistical Analysis

We performed descriptive, group analysis of demographic characteristics with parametric tests, or non-parametric tests for non-normal data, with measures of dispersion as appropriate. Normality was assessed using the Shapiro-Wilk test, visualization of histograms, and Q-Q plots*.* One-way ANOVA was used for comparison of more than 2 time points. Tukey’s multiple comparison test was used for specific post hoc pairwise comparisons. A two-sided *p* < 0.05 was considered to be statistically significant. Data were analyzed using IBM SPSS for Mac (Version 23.0, IBM SPSS Statistics; IBM Corp., Armonk, NY) and GraphPad Prism Version 7.00 (GraphPad Software, La Jolla, CA).

The fraction of time during which the glucose level was below 3.9 mmol/L or above 10 mmol/L was compared for fasting and non-fasting periods. The mean 72-h glucose concentration was calculated and compared within and between groups for both the fasting and non-fasting periods. Glucose variability was determined by calculating MAGE using EasyGV software [22]. The mean increase in interstitial glucose at the time the fast was broken was calculated by subtracting the lowest measured glucose reading during the 30 min preceding breaking of the fast at dusk from the highest reading during the 3 h after breaking of the fast.

## Results

We studied a total of 60 participants enrolled into 4 groups (DO, SGD, SGO, and HC) of 15 participants each.

### Baseline Clinical Characteristics

The 60 participants were comprised of 27 men (45%) and 33 women (55%), aged 21 to 65 years. The surgical groups (SGD and SGO) had a greater proportion of women (63.6%) than the non-surgical groups (DO and HC). As per the study design, HC had lower mean BMI and HbA1c concentration than the other groups (Table 1).

**Table 1:** Characteristics of the different groups at baseline (pre-fasting) and post- fasting (non-fasting) periods

	Sleeve gastrectomy and diabetes (SGD)			Sleeve gastrectomy only (SGO)			T2D-only (DO)			Healthy controls (HC)		
Variable	Baseline	Post-fasting	*p*	Baseline	Post-fasting	*p*	Baseline	Post-fasting	*p*	Baseline	Post-fasting	*p*
Weight (kg)	80.6 (13.5)	80.3 (14.2)	0.562	85.5 (16.9)	86.1 (17.2)	0.199	97.4 (18.0)	97.1 (18.2)	0.396	64.3 (12.8)	64.9 (13.0)	0.208
BMI (kg/m^2^)	31.2 (6.5)	29.9 (5.0)	0.318	30.5 (4.7)	30.7 (4.8)	0.197	34.3 (8.1)	34.2 (8.0)	0.343	23.7 (3.1)	23.9 (3.2)	0.224
Systolic BP (mmHg)	121.8 (17.6)	114.6 (19.3)	0.085	116.4 (8.2)	110.9 (11.8)	0.087	125.3 (12.5)	125.1 (15.5)	0.955	116.5 (13.8)	117.2 (22.5)	0.842
Diastolic BP (mmHg)	71.2 (13.3)	73.9 (11.9)	0.169	67.0 (7.5)	71.6 (6.6)	0.042	74.2 (8.7)	78.3 (10.9)	0.165	67.7 (8.4)	70.4 (8.9)	0.307
HbA1c (%)	6.7 (1.0)	6.5 (0.6)	0.273	5.3 (0.3)	5.2 (0.3)	0.017	8.3 (0.8)	8.0 (0.8)	0.037	5.2 (0.3)	5.1 (0.3)	0.166
Glucose (mmol/L)	6.9 (1.5)	6.6 (1.5)	0.343	5.2 (0.5)	5.1 (0.7)	0.699	9.1 (2.4)	9.2 (1.9)	0.898	4.8 (0.4)	5.2 (0.6)	0.076
Insulin (mU/L)	7.4 (3.7)	8.4 (5.1)	0.422	6.9 (2.9)	7.6 (2.6)	0.468	23.1 (17.2)	15.4 (10.4)	0.043	5.6 (3.1)	8.5 (7.9)	0.206
HOMA-IR	2.3 (1.4)	2.6 (2.2)	0.637	1.6 (0.9)	1.7 (0.7)	0.701	10.0 (7.9)	6.2 (4.4)	0.044	1.2 (0.6)	1.9 (1.6)	0.120
Total Cholesterol (mmol/L)	5.3 (1.2)	5.4 (1.4)	0.667	4.9 (0.7)	4.9 (0.9)	0.940	4.0 (0.6)	4.0(0.8)	0.897	4.5(1.0)	4.2 (0.8)	0.013
HDL-C (mmol/L)	1.5 (0.3)	1.5 (0.3)	0.582	1.4 (0.4)	1.5 (0.3)	0.010	1.1 (0.2)	1.1 (0.2)	0.832	1.3 (0.5)	1.3 (0.4)	0.358
Triglycerides (mmol/L)	1.2 (0.7)	1.6 (1.3)	0.105	0.8 (0.4)	0.8 (0.5)	0.895	1.6 (0.9)	1.5 (0.7)	0.558	0.6 (0.2)	0.9 (0.3)	0.026
LDL-C (mmol/L)	3.2 (1.1)	3.2 (1.3)	0.971	2.7 (0.6)	2.6 (0.5)	0.652	2.3 (0.7)	2.2 (0.7)	0.688	2.7 (0.5)	2.5 (0.6)	0.003

Clinical characteristics of the participants in each group, represented in mean (SD). Data was compared by *t* test, and *p* value ≤ 0.05 was considered as significant difference. T2D: type 2 diabetes; HbA1c: glycated hemoglobin; HOMA-IR: homeostasis model assessment for insulin resistance; HDL-C: high density lipoprotein cholesterol; LDL-C: low density lipoprotein cholesterol

### Glycated Hemoglobin and Insulin Resistance

Compared with baseline (pre-fasting), HbA1c was lower after the fasting period in all 4 groups (Table 1). Homeostasis model assessment of insulin resistance (HOMA-IR) was unchanged in the SGD, SGO, and HC groups after the fasting period but significantly lower in the DO group (*p* < 0.05).

### Mean 72-h Glucose Concentrations

**Fig. 1:**
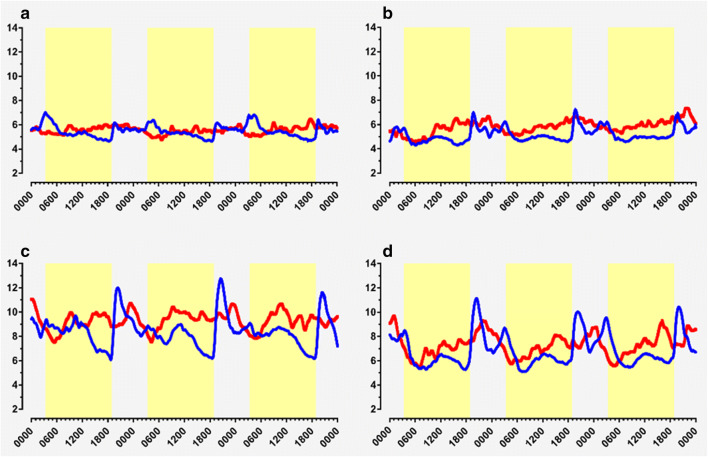
Continuous blood glucose profiles (*y*-axis, mmol/L) over 3 days (*x*-axis, hours) during fasting period (blue trace) and non-fasting period (red trace) in healthy controls (**a**), participants with sleeve gastrectomy without diabetes (**b**), participants with diabetes without bariatric surgery (**c**), and participants with diabetes with sleeve gastrectomy (**d**). Yellow-shaded areas represent hours of daylight/fasting

The mean glucose concentration as determined by CGMS was significantly lower in the fasting period than in the non-fasting period across all 4 groups (Table 2). The highest glucose readings were in the DO group, with the lowest readings seen in the SGO group. ANOVA demonstrated significant differences within and between groups (Supplementary Table 1). The mean glucose profiles of all 4 groups showed a marked increase in blood glucose on breaking the fast at sunset, which was exaggerated in the diabetes (DO and SGD) groups (Fig. 1).

**Table 2:** Results of continuous glucose monitoring parameters in different groups

Groups	Mean 72-h glucose (mmol/L)			Percentage time spent in hyperglycemia*			Percentage time spent in hypoglycemia^†^			MAGE (mmol/L)		
	Fasting	Non-fasting	*p*	Fasting	Non-fasting	*p*	Fasting	Non-fasting	*p*	Fasting	Non-fasting	*p*
SGD	6.4 (5.3–8.0)	6.9 (5.5–8.8)	< 0.001	10.3 (8.7)	14.2 (12.3)	0.291	0.35 (0–2.7)	0.6 (0–3.3)	0.713	5.2 (1.6)	4.5 (1.2)	0.139
SGO	5.0 (4.5–5.5)	5.4 (4.8–6.2)	< 0.001	0 (0)	0 (0)	0.875	3.5 (0.2–10.9)	2.2 (0.4–6.5)	0.252	2.1 (1.7–2.9)	2.0 (1.9–2.3)	0.719
DO	8.1 (6.9–9.7)	9.0 (7.5–10.9)	< 0.001	21.5 (13.3)	35.8 (12.6)	0.008	0 (0–0.6)	0 (0–0.2)	1.00	5.5 (1.7)	6.2 (1.1)	0.143
HC	5.4 (4.8–5.9)	5.5 (4.9–6.2)	0.041	0 (0)	0 (0)	0.250	1.2 (0–2.3)	1.0 (0–7.8)	0.542	2.2 (1.1)	1.9 (0.6)	0.156

Key results in the different groups comparing measures in fasting and non-fasting periods. Data represented in median (interquartile range). *SGD*, Sleeve gastrectomy diabetes; *SGO*, Sleeve gastrectomy only; *DO*, Diabetes only; *HC*, Healthy controls; *MAGE*, Mean amplitude of glycemic excursions

*Hyperglycemia was defined as glucose ≥ 10 mmol/L.

^†^Hypoglycemia was defined as glucose < 3.9 mmol/L.

### Time Spent in Hyperglycemia

In the DO group, participants were hyperglycemic (glucose > 10 mmol/L) for a significantly lower fraction of time during the fasting period than during the non-fasting period (Table 2). Although the trend was similar in the SGD group, it did not reach statistical significance. No readings in the hyperglycemic range were observed in the non-diabetes groups (SGO and HC). Notable differences in the fraction of hyperglycemia time were observed within and between groups (Supplementary Table 2).

### Time Spent in Hypoglycemia

The fraction of time during which people were hypoglycemic (glucose < 3.9 mmol/L) was similar in all groups during the fasting and non-fasting periods (Table 2). However, the SGO group had a higher incidence of hypoglycemia (median, 3.5%) than DO group (both fasting and non-fasting periods, *p* < 0.05 for both), HC (fasting, *p* < 0.05), and the SGD group (fasting, *p* < 0.05). Notable differences were observed within and between groups (Supplementary Table 3).

### Glycemic Variability

MAGE was determined as a measure of glycemic variability and did not differ within each group during the fasting or non-fasting periods (Table 2). Higher glycemic excursions were observed in the SGD group than in the SGO and HC groups (during fasting and non-fasting) (Supplementary Table 4). MAGE was highest in the two diabetes groups (DO and SGD), with significant differences seen between the DO group (during fasting and non-fasting period) and the HC and SGO groups (during fasting and non-fasting periods) (Supplementary Table 4).

**Fig. 2:**
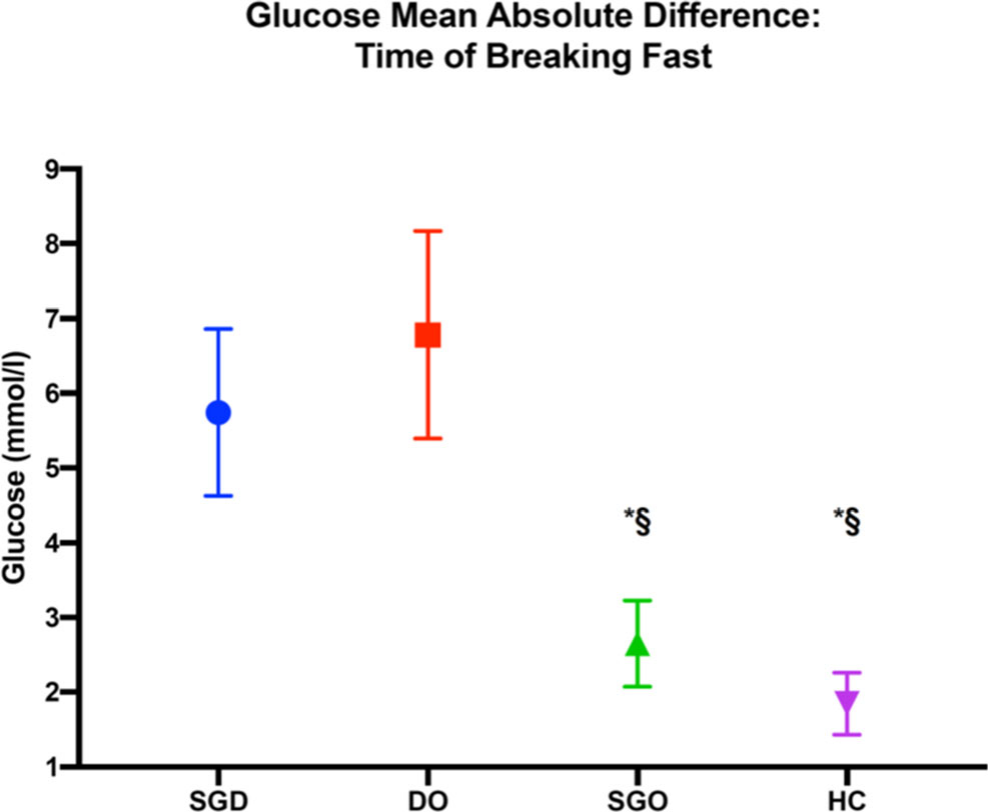
Representation of differences in mean absolute difference in glucose readings at the time of breaking fast. The difference was calculated by subtracting the lowest glucose reading in the 30 min preceding the time of breaking fast from the highest glucose reading in the 3 h after breaking the fast. Error bars represent 95% confidence intervals. SGD: sleeve gastrectomy diabetes; DO: diabetes only; SGO: sleeve gastrectomy only; HC: healthy controls; Group *p < 0.001 when comparing with SGD. Section sign (^§^) indicates *p* < 0.001 when comparing with DO

A marked increase in interstitial glucose was observed at the time of breaking the fast at dusk, particularly in the two diabetes groups (Figs. 1 and 2). The mean absolute differences (MADs) between the lowest glucose reading (during the last 30 min of the fast) and the highest glucose reading (within 3 h of breaking the fast) (mmol/L) were as follows [mean (SD)]: SGD, 5.7 (2.0); DO, 6.8 (2.5); SGO, 2.7 (1.0); and HC, 1.8 (0.7). Differences in MAD were observed among the SGD (5.7 mmol/L), SGO (2.7 mmol/L), and HC (1.8 mmol/L) groups (*p* < 0.001 for all). Similarly, differences were observed among the DO (6.8 mmol/L), SGO (2.7 mmol/L), and HC (1.8 mmol/L) groups (*p* < 0.001 for all). There was no difference in MAD between the two diabetes groups (SGD and DO).

## Discussion

We studied the effects of fasting on glucose excursions in four groups of participants—people with or without diabetes who had or had not undergone sleeve gastrectomy—and report that mean 72-h glucose was lower during fasting period in all the groups. Furthermore, there was reduction in the percentage time spent in the hyperglycemic range in fasting period in the diabetes-only group compared with non-fasting period. Interestingly, there were no differences in the percentage hypoglycemia time in the fasting period compared with non-fasting period. The reduction in the mean 72-h glucose during the fasting period was further supported by a reduction in the follow-up HbA1c readings, which was consistent with other studies [23, 24]. However, despite this reduction in mean glucose, it is important to note the significant rise in the glucose at the time of breaking the fast at dusk in each group. This rise was more pronounced than the rise seen on eating breakfast after an overnight fast in the non-fasting period. After a long daytime fast, the diet consistency and sudden sharp rise in carbohydrate intake are likely to account for this. Traditionally, dates are consumed at the time of breaking fast in Ramadan, and a previous analysis has suggested an average carbohydrate content of 81 g/100 g for dry dates and 55 g/100 g for fresh dates, of which the main sugar is fructose [25]. An excess consumption of dates at the time of breaking the fast in the evening may account for the rapid and exaggerated rise in glucose in all the groups, particularly so in the two diabetes groups. In addition to dates, other calorific sources consumed as part of a large single meal may influence this sharp rise in glucose despite available patient advice sheets advising a more staggered approach to energy intake [26].

This study creates an evidence base (with visual representation) to support such factsheets and can therefore be used to re-emphasize the importance of a measured manner in consuming food after fasting. It confirms that even people with prior sleeve gastrectomy can achieve ample oral carbohydrate intake to result in a rapid rise in systemic glucose levels. This should be avoided because of the risk of post-bariatric surgery dumping syndrome, albeit being less frequent with sleeve gastrectomy [27]. We have previously shown that patients with prior sleeve gastrectomy did not show an increase in adverse symptoms during fasting [28]. Interestingly, the food preference in these patients tended more towards savory rather than sweet foods.

One of the major concerns with fasting is hypoglycemia, and thereby some clinicians advise against fasting, especially in those who have had bariatric surgery. Reassuringly, in the two groups that had sleeve gastrectomy, we did not find an increase in the percentage of time spent in the hypoglycemic range. In addition, there were no episodes of severe hypoglycemia (needing third-party assistance) in any of the groups. This supports the findings of a recent study in which CGMS was done before start of fasting and then at different timepoints during fasting in patients with T2D [29]. Whilst patients on more than two anti-diabetes medications or sulfonylureas showed an increased risk of hypoglycemia in that study [29], a larger study showed no association with sulfonylurea use with increases in hypoglycemia [30]. Another study showed that patients on more than three anti-diabetes medications, especially when physically active, had a higher risk of asymptomatic hypoglycemia during fasting [31]. They suggested that flash glucose monitoring systems could be protective against this, although this does require a level of patient engagement which can limit its efficacy [31]. The advent of “alarmed” glucose monitoring sensors may become tools which can facilitate safe fasting even in patients at higher risk of hypoglycemia and therefore potentially remove lifestyle restrictions that result from diabetes [19, 31].

People without diabetes who had undergone sleeve gastrectomy were at the highest risk of developing hypoglycemia which might suggest that this group of patients should be counseled about this potential risk prior to fasting for prolonged periods and therefore educated on preventing hypoglycemia and the associated symptoms, signs, and treatment. Our findings warrant further studies using different monitoring approaches in this group of patients. In the diabetes groups, there was less percentage time spent in hyperglycemia during fasting compared with the non-fasting period. This contrasts with the findings of Aladawi et al. which showed no changes between fasting and non-fasting periods [29].

In recent years, diets which promote fasting on some days of the week have become increasingly popular for weight-loss, and intermittent fasting has previously been associated with health benefits [32]. The findings of our study lend support to an intermittent fasting approach in people with T2D, even independent of weight changes (there was a non-significant change in weight).

The percentage time spent in a good glycemic range has recently been shown to be a principal glycemic metric, with evidence linking this parameter to microvascular disease outcomes [33]. Whilst we did not find a significant change in this measure, there was an increase in the percentage time in the desired range in the fasting period which needs exploring in further studies.

Mean amplitude of glycemic excursion during the fasting and non-fasting periods in persons with diabetes with and without sleeve gastrectomy was higher than in the non-diabetes groups, but the range of values were not dissimilar to those in published series on fasting participants with T2D [34, 35]. However, the lower MAGE in the diabetes-only group during fasting period probably reflects better glycemic control during fasting [35].

The findings of our study can be translated into clinical practice in a number of ways. Firstly, we show that patients after sleeve gastrectomy do not show marked increases in hypoglycemia during fasting which may alleviate some physician- and patient-associated anxiety around fasting. However, conversely, we display that an absence of a history of diabetes does not alleviate hypoglycemia risk in patients after sleeve gastrectomy and therefore appropriate preventative education should be delivered to all patients wishing to fast after bariatric surgery. Secondly, we demonstrate that, overall, there are favorable glycemia-associated changes during fasting and so an intermittent fasting lifestyle approach can be beneficial in selected patients. Thirdly, this study shows that in different groups, the rise in blood glucose at the time of breaking the fast is substantial—this may be due to heightened energy intake (especially simple sugars) but can represent physiological adaptations to fasting. It is therefore imperative that patients are educated on staggering their intake of carbohydrates and calories to prevent marked glucose excursions.

### Strengths and Limitations

This is the first study to our knowledge to examine glucose fluctuations in response to fasting in a four-way analysis of persons with or without diabetes with or without sleeve gastrectomy. The design of the study allowed robust comparisons within as well as across groups. Although power and sample size estimations were not applicable as a novel study of its type, the strongly significant results for the primary endpoint of mean 72-h glucose levels in fasting versus non-fasting periods across all study groups confirm adequacy of the sample size. Most people after sleeve gastrectomy go into remission of T2D. We selected a subgroup who did not go into remission of T2D after surgery, probably representing more advanced disease. However, it was reassuring that even these patients, who were also treated with anti-diabetic medications, did not have increased episodes of hypoglycemia.

## Conclusion

We conclude that, compared with healthy controls, interstitial glucose measured by CGMS during intermittent fasting in selected patients with T2D, and patients with and without T2D at least 1 year after sleeve gastrectomy, did not show significant differences or hypoglycemia.

## Electronic supplementary material

ESM 1 (DOCX 47 kb)
