# Supplementary material for: Continuous Glucose Monitoring of Glycemic Variability During Fasting Post-Sleeve Gastrectomy
Source: Obes Surg. 2020 Jul 17;30(10):3721–9. doi: 10.1007/s11695-020-04505-4 (PMC7467959; doi:10.1007/s11695-020-04505-4)
Supplement: Supplementary file 1 — (DOCX 47 kb) [file 11695_2020_4505_MOESM1_ESM.docx]

**Supplementary Information**

**Supplementary table 1.** Comparison of mean 72-hour CGM glucose value during fasting and non-fasting period.

| Group | DO (Fasting) | DO (Non-fasting) | HC (Fasting) | HC (Non-fasting) | SGD (Fasting) | SGD **(**Non-fasting**)** | SGO (Fasting) | SGO (Non-fasting) |
| --- | --- | --- | --- | --- | --- | --- | --- | --- |
| DO (Fasting) |  | -0.8  (0.0)  **** | 3.0  (0.0)  **** | 2.9  (0.0)  **** | 1.6  (0.0)  **** | 1.2  (0.0)  **** | 3.4  (0.0)  **** | 2.7  (0.0)  **** |
| DO (Non-fasting) | 0.8  (0.0)  **** |  | 3.8  (0.0)  **** | 3.7  (0.0)  **** | 2.4  (0.0)  **** | 2.0  (0.0)  **** | 4.2  (0.0)  **** | 3.5  (0.0)  **** |
| HC (Fasting) | -3.0  (0.0)  **** | -3.8  (0.0)  **** |  | -0.1  (0.0)  * | -1.4  (0.0)  **** | -1.9  (0.0)  **** | 0.4  (0.0)  **** | -0.3  (0.0)  **** |
| HC (Non-fasting) | -2.9  (0.0)  **** | -3.7  (0.0)  **** | 0.1  (0.0)  * |  | -1.3  (0.0)  **** | -1.8  (0.0)  **** | 0.4  (0.0)  **** | -0.2  (0.0)  **** |
| SGD (Fasting) | -1.6  (0.0)  **** | -2.4  (0.0)  **** | 1.4  (0.0)  **** | 1.3  (0.0)  **** |  | -0.5  (0.0)  **** | 1.7  (0.0)  **** | 1.1  (0.0)  **** |
| SGD (Non-fasting) | -1.2  (0.0)  **** | -2.0  (0.0)  **** | 1.9  (0.0)  **** | 1.8  (0.0)  **** | 0.5  (0.0)  **** |  | 2.2  (0.0)  **** | 1.6  (0.0)  **** |
| SGO (Fasting) | -3.4  (0.0)  **** | -4.2  (0.0)  **** | -0.4  (0.0)  **** | -0.4  (0.0)  **** | -1.7  (0.0)  **** | -2.2  (0.0)  **** |  | -0.7  (0.0)  **** |
| SGO (Non-fasting) | -2.7  (0.0)  **** | -3.5  (0.0)  **** | 0.3  (0.0)  **** | 0.2  (0.0)  **** | -1.1  (0.0)  **** | -1.6  (0.0)  **** | 0.7  (0.0)  **** |  |

Data (mmol/L) are presented as the difference between the means (standard error of mean). Differences are shown as the difference between the mean value of the group in the horizontal column vs. the vertical column. For example, the difference between the HC (Non-fasting) group and the DO (Fasting) group was −2.9 mmol/L, indicating a higher mean CGM glucose concentration in the latter group. DO, diabetes only; HC, healthy controls; SGD: sleeve gastrectomy diabetes; SGO: sleeve gastrectomy only.

****p < 0.0001; ***p < 0.001; **p < 0.01; *p < 0.05; ns, no significant difference.

**Supplementary table 2.** Comparison of mean 72-hour CGM percentage hyperglycaemia in fasting and non-fasting period.

| Group | DO (Fasting) | DO (Non-fasting) | HC (Fasting) | HC (Non-fasting) | SGD (Fasting) | SGD **(**Non-fasting**)** | SGO (Fasting) | SGO (Non-fasting) |
| --- | --- | --- | --- | --- | --- | --- | --- | --- |
| DO (Fasting) |  | -14.3  (3.1)  *** | 21.0  (3.1)  **** | 21.4  (3.1)  **** | 11.2  (3.1)  ** | 7.3  (3.1)  ns | 21.3  (3.1)  **** | 21.4  (3.1)  **** |
| DO (Non-fasting) | 14.3  (3.1)  *** |  | 35.2  (3.1)  **** | 35.7  (3.1)  **** | 25.5  (3.1)  **** | 21.6  (3.1)  **** | 35.6  (3.1)  **** | 35.7  (3.1)  **** |
| HC (Fasting) | -21.0  (3.1)  **** | -35.2  (3.1)  **** |  | 0.5  (3.1)  ns | -9.8  (3.1)  * | -13.6  (3.1)  *** | 0.31  (3.1)  ns | 0.4  (3.1)  ns |
| HC (Non-fasting) | -21.4  (3.1)  **** | -35.7  (3.1)  **** | -0.5  (3.1)  ns |  | -10.3  (3.1)  * | -14.1  (3.1)  *** | -0.2  (3.1)  ns | -0.1  (3.1)  ns |
| SGD (Fasting) | -11.2  (3.1)  ** | -25.5  (3.1)  **** | 9.8  (3.06)  * | 10.3  (3.1)  * |  | -3.9  (3.1)  ns | 10.1  (3.1)  * | 10.2  (3.1)  * |
| SGD (Non-fasting) | -7.3  (3.1)  ns | -21.6  (3.1)  **** | 13.6  (3.12)  *** | 14.1  (3.1)  *** | 3.9  (3.1)  ns |  | 13.9  (3.1)  *** | 14.1  (3.2)  *** |
| SGO (Fasting) | -21.3  (3.1)  **** | -35.6  (3.1)  **** | -0.3  (3.06)  ns | 0.2  (3.1)  ns | -10.1  (3.7)  * | -13.9  (3.1)  *** |  | 0.1  (3.1)  ns |
| SGO (Non-fasting) | -21.4  (3.1)  **** | -35.7  (3.1)  **** | -0.4  (3.1)  ns | 0.1  (3.1)  ns | -10.2  (3.1)  * | 14.1  (3.1)  *** | -0.1  (3.1)  ns |  |

Hyperglycemia defined as glucose ≥10 mmol/L. Data (%) are presented as the difference between means (standard error of mean). Differences are shown as the difference between the mean in the horizontal column vs. the vertical column. For example, the difference between the HC (Non-fasting) group and the DO (Fasting) group was −21.4%, indicating a lower value in the latter group.

DO, diabetes only; HC, healthy controls; SGD: sleeve gastrectomy diabetes; SGO: sleeve gastrectomy only.

****p < 0.0001; ***p < 0.001; **p < 0.01; *p < 0.05; ns, no significant difference.

**Supplementary table 3.** Comparison of mean 72-hour CGM percentage hypoglycaemia time (over 72 hours) in fasting and non-fasting period.

| Group | DO (Fasting) | DO (Non-fasting) | HC (Fasting) | HC (Non-fasting) | SGD (Fasting) | SGD **(**Non-fasting**)** | SGO (Fasting) | SGO (Non-fasting) |
| --- | --- | --- | --- | --- | --- | --- | --- | --- |
| DO (Fasting) |  | -0.2  (1.6)  ns | -1.7  (1.6)  ns | -3.4  (1.7)  ns | -2.7  (1.6)  ns | -1.2  (1.6)  ns | -7.02  (1.6)  ** | -3.3  (1.6)  ns |
| DO (Non-fasting) | 0.2  (1.6)  ns |  | -1.5  (1.6)  ns | -3.3  (1.7)  ns | -2.5  (1.6)  ns | -1.0  (1.7)  ns | -6.8  (1.6)  ** | -3.1  (1.6)  ns |
| HC (Fasting) | 1.7  (1.6)  ns | 1.5  (1.6)  ns |  | -1.8  (1.7)  ns | -1.0  (1.6)  ns | 0.5  (1.7)  ns | -5.4  (1.6)  * | -1.6  (1.6)  ns |
| HC (Non-fasting) | 3.5  (1.7)  ns | 3.3  (1.7)  ns | 1.8  (1.7)  ns |  | 0.8  (1.7)  ns | 2.3  (1.7)  ns | -3.6  (1.7)  ns | 0.1  (1.7)  ns |
| SGD (Fasting) | 2.7  (1.6)  ns | 2.5  (1.6)  ns | 1.0  (1.6)  ns | -0.8  (1.7)  ns |  | 1.5  (1.7)  ns | -4.3  (1.6)  ns | -0.6  (1.6)  ns |
| SGD (Non-fasting) | 1.2  (1.6)  ns | 1.0  (1.7)  ns | -0.5  (1.7)  ns | -2.3  (1.7)  ns | -1.5  (1.7)  ns |  | -5.8  (1.7)  * | -2.1  (1.7)  ns |
| SGO (Fasting) | 7.0  (1.6)  ** | 6.8  (1.6)  ** | 5.4  (1.6)  * | 3.6  (1.7)  ns | 4.3  (1.6)  ns | 5.8  (1.7)  * |  | 3.7  (1.6)  ns |
| SGO (Non-fasting) | 3.3  (1.6)  ns | 3.1  (1.6)  ns | 1.6  (1.6)  ns | -0.1  (1.7)  ns | 0.6  (1.6)  ns | 2.1  (1.7)  ns | -3.7  (1.6)  ns |  |

Hypoglycemia defined as a glucose concentration <3.9 mmol/L. Data (%) are presented as the difference between means (standard error of mean). Differences are shown as the difference between the mean value of the group in the horizontal column vs. the vertical column. For example, the difference between the HC (Non-fasting) group and the DO (Non-fasting) group was 3.5, indicating a smaller value in the latter group.

DO, diabetes only; HC, healthy controls; SGD: sleeve gastrectomy diabetes; SGO: sleeve gastrectomy only.

****p < 0.0001; ***p < 0.001; **p < 0.01; *p < 0.05; ns, no significant difference.

**Supplementary table 4.** Post-hoc multiple comparison of mean amplitude of glycemic excursion (MAGE) value between the group of fasting and non-fasting period**.**

| Tukey's multiple comparisons test  First group vs. Second group | Mean of first group | Mean of second group | Mean Difference | 95% CI | Adjusted p value* |
| --- | --- | --- | --- | --- | --- |
| DO (Fasting) vs. DO (Non-fasting) | 5.5 | 6.2 | -0.7 | -2.1, 0.6 | ns |
| DO (Fasting) vs. HC (Fasting) | 5. 5 | 2.2 | 3.3 | 1.9, 4.6 | <0.001 |
| DO (Fasting) vs. HC (Fasting) | 5. 5 | 1.9 | 3.6 | 2.2, 5.0 | <0.001 |
| DO (Fasting) vs. SGD (Fasting) | 5.5 | 5.2 | 0.3 | -1.0, 1.6 | ns |
| DO (Fasting) vs. SGD (Non-fasting) | 5.5 | 4.5 | 1.0 | -0.4, 2.3 | ns |
| DO (Fasting) vs. SGO (Fasting) | 5.5 | 2.4 | 3.1 | 1.8, 4.4 | <0.001 |
| DO (Fasting) vs. SGO (Non-fasting) | 5.5 | 2.2 | 3.3 | 1.9, 4.6 | <0.001 |
| DO (Non-fasting) vs. HC (Fasting) | 6.2 | 2.2 | 4.0 | 2.7, 5.3 | <0.001 |
| DO (Non-fasting) vs. HC (Non-fasting) | 6.2 | 1.9 | 4.3 | 2.9, 5.7 | <0.001 |
| DO (Non-fasting) vs. SGD (Fasting) | 6.2 | 5.2 | 1.0 | -0.3, 2.4 | ns |
| DO (Non-fasting) vs. SGD (Non-fasting) | 6.2 | 4.5 | 1.7 | 0.35, 3.1 | 0.004 |
| DO (Non-fasting) vs. SGO (Fasting) | 6.2 | 2.4 | 3.8 | 2.5, 5.2 | <0.001 |
| DO (Non-fasting) vs. SGO (Non-fasting) | 6.2 | 2.2 | 4.0 | 2.7, 5.3 | <0.001 |
| HC (Fasting) vs. HC (Non-fasting) | 2.2 | 1.9 | 0.3 | -1.1, 1.7 | ns |
| HC (Fasting) vs. SGD (Fasting) | 2.2 | 5.2 | -3.0 | -4.3, -1.6 | <0.001 |
| HC (Fasting) vs. SGD (Non-fasting) | 2.2 | 4.5 | -2.3 | -3.7, -0.9 | <0.001 |
| HC (Fasting) vs. SGO (Fasting) | 2.2 | 2.4 | -0.2 | -1.5, 1.2 | ns |
| HC (Fasting) vs. SGO (Non-fasting) | 2.2 | 2.2 | -0.0 | -1.3, 1.3 | ns |
| HC (Non-fasting) vs. SGD (Fasting) | 1.9 | 5.2 | -3.3 | -4.7, -1.9 | <0.001 |
| HC (Non-fasting) vs. SGD (Non-fasting) | 1.9 | 4.5 | -2.6 | -4.1, -1.2 | <0.001 |
| HC (Non-fasting) vs. SGO (Fasting) | 1.9 | 2.4 | -0.5 | -1.9, 0.9 | ns |
| HC (Non-fasting) vs. SGO (Non-fasting) | 1.9 | 2.2 | -0.3 | -1.7, 1.0 | ns |
| SGD (Fasting) vs. SGD (Non-fasting) | 5.2 | 4.5 | 0.7 | -0.7, 2.0 | ns |
| SGD (Fasting) vs. SGO (Fasting) | 5.2 | 2.4 | 2.8 | 1.4, 4.1 | <0.001 |
| SGD (Fasting) vs. SGO (Non-fasting) | 5.2 | 2.2 | 3.0 | 1.6, 4.3 | <0.001 |
| SGD (Non-fasting) vs. SGO (Fasting) | 4.5 | 2.4 | 2.1 | 0.8, 3.5 | 0.001 |
| SGD (Non-fasting) vs. SGO (Non-fasting) | 4.5 | 2.2 | 2.3 | 0.9, 3.6 | <0.001 |
| SGO (Fasting) vs. SGO (Non-fasting) | 2.4 | 2.2 | 0.2 | -1.2, 1.5 | ns |

DO: Diabetes only; HC: Healthy Controls; SGD: Sleeve Gastrectomy Diabetes; SGO: Sleeve Gastrectomy only, *ANOVA test was used for the significant differences. ns: non-significant
